# Disease-free survival of 15 years after primary surgery in a patient with advanced high-grade serous ovarian cancer: a case report and literature review

**DOI:** 10.3389/fonc.2025.1468196

**Published:** 2025-01-27

**Authors:** Yaoqi Shi, Shuaiying Zhu, Jiangjing Shan, Yuhong Xu

**Affiliations:** Department of Gynecology, Shaoxing People’s Hospital, Shaoxing, Zhejiang, China

**Keywords:** long-term survivors, prognosis, high-grade serous ovarian cancer, case report, review

## Abstract

**Background:**

Ovarian cancer, particularly high-grade serous ovarian cancer (HGSOC), is the most lethal gynecological tumor, with most patients experiencing recurrence within 5 years. Long-term survival in HGSOC patients with advanced stages is exceedingly rare.

**Case summary:**

We report a case of advanced HGSOC with exceptional long-term recurrence-free survival following initial treatment. In June 2009, the patient underwent suboptimal cytoreductive surgery for stage IIIC ovarian cancer, including total hysterectomy, bilateral salpingo-oophorectomy, omentectomy, appendectomy, and resection of mesenteric and peritoneal lesions. Postoperatively, residual lesions were observed in the mesenteries and para-aortic lymph nodes. Despite unfavorable prognostic factors (advanced stage, aggressive pathology, and incomplete resection), the patient showed remarkable chemosensitivity, remaining recurrence-free for 15 years.

**Conclusion:**

The factors influencing long-term survival in HGSOC patients are not yet fully understood. We present this rare case to contribute data for further studies on long-term survival in advanced HGSOC.

## 1 Introduction

Ovarian cancer has long been recognized as the most lethal gynecological malignancy. According to global cancer statistics, there were 324,398 new cases and 206,839 deaths due to ovarian cancer in 2022 (1). Despite advancements in surgical techniques and maintenance therapies, the 5-year survival rate for ovarian cancer patients in some countries remains below 40% (2). Because of the poor prognosis, patients with ovarian cancer are also usually not followed for long periods of time. High-grade serous ovarian cancer (HGSOC) is the most common type of epithelial ovarian cancer (EOC) (3). Although HGSOC initially responds well to treatment, it often develops resistance to chemotherapy over time, leading to patient mortality. However, a small subset of patients with advanced HGSOC exhibit an exceptional response to treatment, with some achieving long-term survival without disease recurrence (4–9). Long-Term Survivors (LTS) are typically defined as patients who survive for at least 10 years after diagnosis, constituting a heterogeneous population with slightly varying definitions in each study (10). The reasons behind the extended survival of these patients remain unclear, but unraveling their distinctive characteristics may offer insights for individuals with shorter life expectancies. This article presents a case of a patient with advanced HGSOC who, despite having a surgical residual lesion greater than 1 cm and not undergoing maintenance therapy, remained disease-free for 15 years following the initial treatment. It also reviews the existing literature regarding long-term survival in HGSOC patients.

## 2 Case presentation

A 46-year-old woman presented to our hospital in June 2009 with a palpable mass in her lower abdomen. Ultrasonography revealed a large solid-cystic mass in the pelvis. At that time, the patient had no menstrual changes, abdominal pain, distension, or bowel or urinary discomfort. Apart from a history of cesarean section, she was in good health with no history of smoking or drinking. Contrast-enhanced computed tomography (CT) of the entire abdomen showed a substantial mass in the lower abdomen (**Supplementary Figure 1A**) and enlarged lymph nodes near the abdominal aorta (**Supplementary Figure 1B**). Hematological tests indicated elevated tumor marker levels, including a CA125 level of 4014.7 U/mL (reference value: <35 U/mL).

Considering the possibility of a malignant tumor in the pelvic-abdominal cavity, the patient underwent surgery on June 29, 2009. During intraoperative exploration, 200ml of light red fluid was discovered in the pelvic cavity. The left ovarian tumor measured 12 ×10 ×10 cm, displaying an uneven surface and a multilocular appearance, resembling cauliflower with a crisp paper-like texture and extensive blood invasion. The tumor was tightly adhered to the greater omentum, mesenteric root, and sigmoid colon. Two 3 ×4 cm masses were identified in the uterorectal fossa, exhibiting a brittle texture. Scattered nodules of various sizes were present on the pelvic peritoneum, mesentery, and greater omentum, along with 2 enlarged lymph nodes measuring 2 ×1.5 cm near the abdominal aorta. The uterus was anteriorly positioned and normal in size, while the right ovary, fallopian tube, and appendix appeared normal. Intraoperative rapid freezing indicated ovarian epithelial malignancy, leading to the removal of her uterus, bilateral adnexa, appendix, greater omentum, peritoneal metastases, and portions of mesenteric metastases. Unfortunately, the surgery did not remove the enlarged para-aortic lymph nodes. Additionally, 80 mg of cisplatin was administered into the abdominal cavity for chemotherapy. Postoperative pathological examination revealed poorly differentiated serous adenocarcinoma of the left ovary, with cancer tissue infiltrating the lesions in the retroperitoneum and mesentery root. Postoperative pathologic findings suggest a HGSOC in left ovary (**Supplementary Figure 2**).

The patient recovered well after surgery. Based on the postoperative pathology results, she was staged IIIC according to the FIGO 1988 classification. She underwent 7 cycles of intravenous chemotherapy with paclitaxel combined with cisplatin, normalizing her CA125 levels after 3 courses (**Supplementary Figure 3**). The patient responded well to chemotherapy with no significant adverse effects. Over the next 15 years of follow-up, including the latest in October 2024, the patient’s tumor markers remained normal. At the latest follow-up, the patient’s abdominal enhanced CT examination did not suggest any disease recurrence (**Supplementary Figure 4**). She maintains good health, does not smoke or drink, and has no mental illnesses such as depression or anxiety. Unfortunately, the patient refused a genetic testing due to financial reasons.

## 3 Discussion

Despite the effectiveness of surgery, combination chemotherapy, and targeted therapy in achieving disease remission for most patients with advanced ovarian cancer, the majority still experience recurrence within three years, with a 10-year disease-free survival rate of less than 10-30% (8). As the most common and aggressive subtype of ovarian cancer, HGSOC is particularly lethal among gynecological malignancies, with 75% of patients dying within 10 years of initial diagnosis (11). While most HGSOC tumors are initially responsive to chemotherapy, resistance typically develops after multiple relapses, leading to treatment failure and patient mortality (12). Therefore, identifying factors associated with long-term survival in ovarian cancer patients is of paramount importance.

Based on existing research, the prognosis of ovarian cancer patients is often influenced by factors such as optimal surgical cytoreduction, primary platinum sensitivity, young age, good physical status, and relatively good clinicopathological factors, all of which are also associated with long-term survival (13, 14). Andreas du Bois et al. (15) analyzed 3,126 patients with advanced ovarian cancer and found that a tremendous impact of surgical outcome after initial debulking surgery with both impact on progression-free and overall survival. Complete surgical debulking improves prognosis in any stage in advanced ovarian cancer. A study utilized a dataset of patients with stage III epithelial ovarian cancer or primary peritoneal cancer who underwent optimal tumor debulking surgery in GOG-104, 114, and 172 trials. By conducting Cox regression survival analysis, it was revealed that young age was an independent prognostic factor for long-term disease free survival exceeding 10 years. This study proposes that the impact of age may be attributed to the absence of comorbidities or to the tumor biology characteristics of younger patients who have a higher likelihood of BRCA mutations (16). Normal CA125 levels before surgery or platinum treatment are also indicative of a more favorable prognosis (17, 18). Some studies have shown that genomic instability leads to a better prognosis and mutations in specific genes significantly improve prognosis (14, 19). Dale W. Garsed et al. (14) analyzed 60 patients with HGSOC and found that concurrent mutations in RB1 and BRCA1 may be associated with a good treatment response and outcome. In the subgroup of this study, there were mixed defects in BRCA1 and BRCA2, which demonstrated the longest progression-free survival despite having a high percentage of postoperative residual disease after surgery (R > 1), suggesting that BRCA1 combined with BRCA2-deficient tumors are particularly sensitive to platinum-based chemotherapy. Poly(ADP-ribose) polymerase (PARP) inhibitors have been approved as first-line maintenance therapy following platinum-based chemotherapy. Recent clinical trials, including SOLO-1 (20), PAOLA-1 (21), PRIMA (22), and ATHENA-mono phase III (23), have demonstrated that PARP inhibitors significantly improve progression-free survival (PFS) and overall survival (OS) in patients with BRCA mutation-positive ovarian cancer. Long-term follow-up data from these studies further indicate that PARP inhibitors are effective in enhancing long-term survival in this patient population. Zhao et al. (24) identified 488 significantly dysregulated genes through transcriptomic data from six independent studies of HGSOC in the NCBI GEO database, of which 232 were significantly associated with OS. These genes are significantly enriched in processes such as the p53 signaling pathway, cell cycle division, epithelial cell differentiation, and vascular system development. They established a prognostic scoring system for 11 genes (RAD51AP1, CADPS2, DSE, ITGB8, PDE10A, GALNT10, SNX1, MTHFD2, C9orf16, PYCR1, and ARL4) in HGSOC patients, which robustly predicted OS in 100 sampled test sets. The correlation between immune factors and LTS of HGSOC patients has been observed in recent years. Evaluating the combined counts of CD103+ and CD3+ can enhance the prognostic value of tumor infiltrating lymphocytes (TIL) and predict long-term survival based on the type and degree of the immune response (25). Intraepithelial CD4+ cells are positively correlated with improved PFS and OS in patients with HGSOC, whereas epithelial CD8+ cells are associated with increased PFS (26). Moreover, PD-L1 expression is believed to be associated with the long-term survival in HGSOC (27, 28). Higher PD-L1 positive stromal TIL scores were associated with lower survival rates. Currently, several antibody-drug conjugates (29) and immune checkpoint inhibitors (30) for ovarian cancer are undergoing clinical trials. However, there remains a lack of trial data regarding the impact of immunotherapy on long-term survival. Certain lifestyle habits can also affect the prognosis. Not smoking as well as higher physical activity can improve the prognosis of ovarian cancer patients (31, 32). Exposure to cigarette smoke may adversely affect systemic immunity and the tumor immune response, and is associated with an increased risk of ovarian cancer (33). The survival outcomes of women with HGSOC due to BRCA1 deficiency may be influenced by mutagen exposure, such as smoking and/or exposure to acetaldehyde (14). Physical activity is beneficial for enhancing the quality of life in ovarian cancer patients (34). Furthermore, women who exhibited moderate to high dietary quality prior to diagnosis experienced significantly lower mortality rates from HGSOC (35). Adherence to the Alternate Mediterranean Diet (36), along with increased intake of dietary fiber, carbohydrates (37), and fish and seafood (38), has the potential to improve the prognosis for ovarian cancer patients. Conversely, a decline in dietary quality following diagnosis is associated with reduced survival rates in ovarian cancer (39). Utilizing appropriate emotion-focused coping strategies and having strong social support also appear to improve prognosis (40).

In this case, the patient’s preoperative CA125 levels showed a significant increase but did not return to normal before chemotherapy. The initial operation did not include resection of enlarged para-aortic lymph nodes and a portion of abdominal metastases. The maximum diameter of the residual tumor is greater than 10 mm (R > 1). After finishing the chemotherapy, the patient was not maintained on the targeted drug. During the next 15 years of follow-up, the patient’s serum CA125 levels and abdominal CT findings continued to remain normal with no evidence of tumor recurrence. It is extremely rare for this patient to achieve a disease-free survival of 15 years despite having so many unfavorable prognostic factors. There is limited research on LTS of HGSOC, and only a few cases of relapse have been reported more than 10 years after the initial treatment. Here are cases of HGSOC in which relapses occurred or no recurrence was observed for more than 10 years after the initial treatment (**Table 1**). It should be noted that the case we reported is similar to previously documented patients with BRCA mutations in the literature. Patients with BRCA1/2 mutations are typically diagnosed at a younger age, often present at an advanced stage, predominantly exhibit HGSOC as the histological type (41), and demonstrate increased sensitivity to platinum-based chemotherapy (42). All of these characteristics align with the details of the reported case. Unfortunately, due to financial constraints, this patient declined genetic testing, resulting in the inability to determine the genetic mutation. The inability to analyze the reasons for this patient’s long-term survival at the genetic level is a shortcoming of this study. Long disease-free survival after initial treatment in HGSOC may depend on rare events or the interaction of multiple factors, such as extreme sensitivity to initial chemotherapy or enhanced anti-cancer immunity. Analyzing these patients may help to identify clinicopathological factors and biological indicators that are useful for predicting prognosis and potentially limiting the development of chemotherapy drug resistance. HGSOC patients with adverse prognostic factors may also become LTS, and current research cannot predict who will be LTS based on individual clinical and pathological characteristics. We present this case in order to explore the possibility of collaborating with multiple research centers to conduct a study on LTS of HGSOC. By including multiple long-term survival patients in the same cohort, it may be possible to identify additional factors that influence the long-term survival of patients with HGSOC and to discover biomarkers that can predict which individuals are likely to achieve long-term survival. This could establish a theoretical foundation for enhancing the survival rate of ovarian cancer patients.

**Table 1:** Reports of HGSOC with a recurrence interval or no recurrence of more than 10 years after initial treatment.

Author	Publication year	Number of Cases	Diagnosis age	Stage	Surgical status	Genomic testing	Targeted therapy	Relapse interval (years)	Initial treatment	Chemotherapy	Recurrence location
Benjamin Zylberberg (4)	2004	1	50	IC	NA	NA	NA	21	Total abdominal hysterectomy, bilateral salpingo-oophorectomy, omentectomy, adjuvant chemotherapy	NA	Axillary nodule
Kunihiko Izuishi (6)	2010	1	32	IIC	NA	NA	NA	20	Total abdominal hysterectomy, bilateral salpingo-oophorectomy, adjuvant chemotherapy, a year later, second look surgery, low anterior resection, and adjuvant chemotherapy	3 courses of initial combination chemotherapy with 5-fluorouracil, cyclophosphamide, adriamycin, and cisplatin, second adjuvant chemotherapy unknown	Spleen
Fanny Dao (9)	2016	88	NA	NA	78 optimal	NA	NA	No Recurrences	NA	Mainly platinum-based chemotherapy or neoadjuvant chemotherapy, course not specified	No Recurrences
Kosei Takagi (5)	2021	1	46	NA	NA	NA	NA	30	Radical total hysterectomy, bilateral adnexectomy, adjuvant chemotherapy	NA	Para-aortic and Virchow lymph nodes, Spleen, Intestine
Takashi Mitamura (8)	2022	4	47/52/73/56	III/III/III/IV	All optimal	1 germline BRCA1 mutation	NA	11/11/13/14	NA	NA	NA
Yoko Suzuki (7)	2024	1	57	IC	NA	Refusal	NA	18	Hysterectomy, bilateral salpingo-oophorectomy, subtotal omentectomy, adjuvant chemotherapy	5 courses of paclitaxel-carboplatin combination chemotherapy	Liver, Spleen
Present case	2024	1	46	IIIC	R>1	Refusal	NO	No Recurrences	hysterectomy, bilateral salpingo-oophorectomy, appendectomy, omentectomy, resection of abdominal metastatic leisons, adjuvant chemotherapy	7 courses of paclitaxel-carboplatin combination chemotherapy	No Recurrences

## 4 Conclusions

Long-term surviving patients with advanced ovarian cancer are exceedingly rare. Prognostic factors that impact ovarian cancer patients include tumor stage, initial surgery, chemotherapy sensitivity, genetic mutation status, lifestyle, and immune factors. Here, we present a rare case of HGSOC with an exceptionally long period of disease-free survival. Despite having advanced disease stage, high tumor malignancy, suboptimal tumor cytoreduction, elevated CA125 levels before chemotherapy, and receiving no maintenance therapy, this patient remained free of recurrence for 15 years following initial treatment. Our report aims to contribute valuable data to studies on long-term disease-free survival in advanced HGSOC. In the future, we aim to further explore the genetic, lifestyle, and immune status of ovarian cancer patients through collaborative efforts across multiple centers. This will help identify potential influencing factors and offer new insights to enhance the prognosis of advanced ovarian cancer.

## Data Availability

The original contributions presented in the study are included in the article/**Supplementary Material**. Further inquiries can be directed to the corresponding author.

## Supplementary material

Supplementary Figure 1 Contrast-enhanced computed tomography (CT) images of the patient's abdomen (Date: 2009-6-27). **(A)** Preoperative tumors in the abdominal cavity. Enhanced CT findings suggest a large cystic solid mass in the lower abdomen as well as an encapsulated effusion adjacent to the mass. **(B)** Preoperative enlarged para-aortic abdominal lymph nodes. Enhanced CT showed enlarged lymph nodes adjacent to the abdominal aorta. Intraoperative exploration here revealed two lymph nodes measuring approximately 2*1.5 cm.

Supplementary Figure 2 Histopathologic findings of representative left ovarian high-grade serous ovarian cancer with multiple metastases (HE staining, ×200, scale bars: 200 µm). **(A)** Diffusely infiltrating tumor in the left ovary. **(B)** Metastatic tumor lesions in the mesentery. **(C)** Metastatic tumor lesions in the posterior peritoneum.

Supplementary Figure 3 Serum CA125 levels in this patient during the 15 years. The patient's CA125 level turned normal (reference value: <35 U/mL) after 3 chemotherapy treatments, and never elevated again during the subsequent 15 years of follow-up. (NRM=normal; 1st chemo= before the first chemotherapy).

Supplementary Figure 4 Contrast-enhanced CT images of the patient's abdomen (Date:2024-10-10). **(A)** No tumour recurrence seen in the abdominal cavity. **(B)** Para-abdominal aortic lymph nodes are not enlarged.
