# Supplementary figures and images for: Disease-free survival of 15 years after primary surgery in a patient with advanced high-grade serous ovarian cancer: a case report and literature review
Source: Front Oncol. 2025 Jan 27;15:1468196. doi: 10.3389/fonc.2025.1468196 (PMC11807797; doi:10.3389/fonc.2025.1468196)

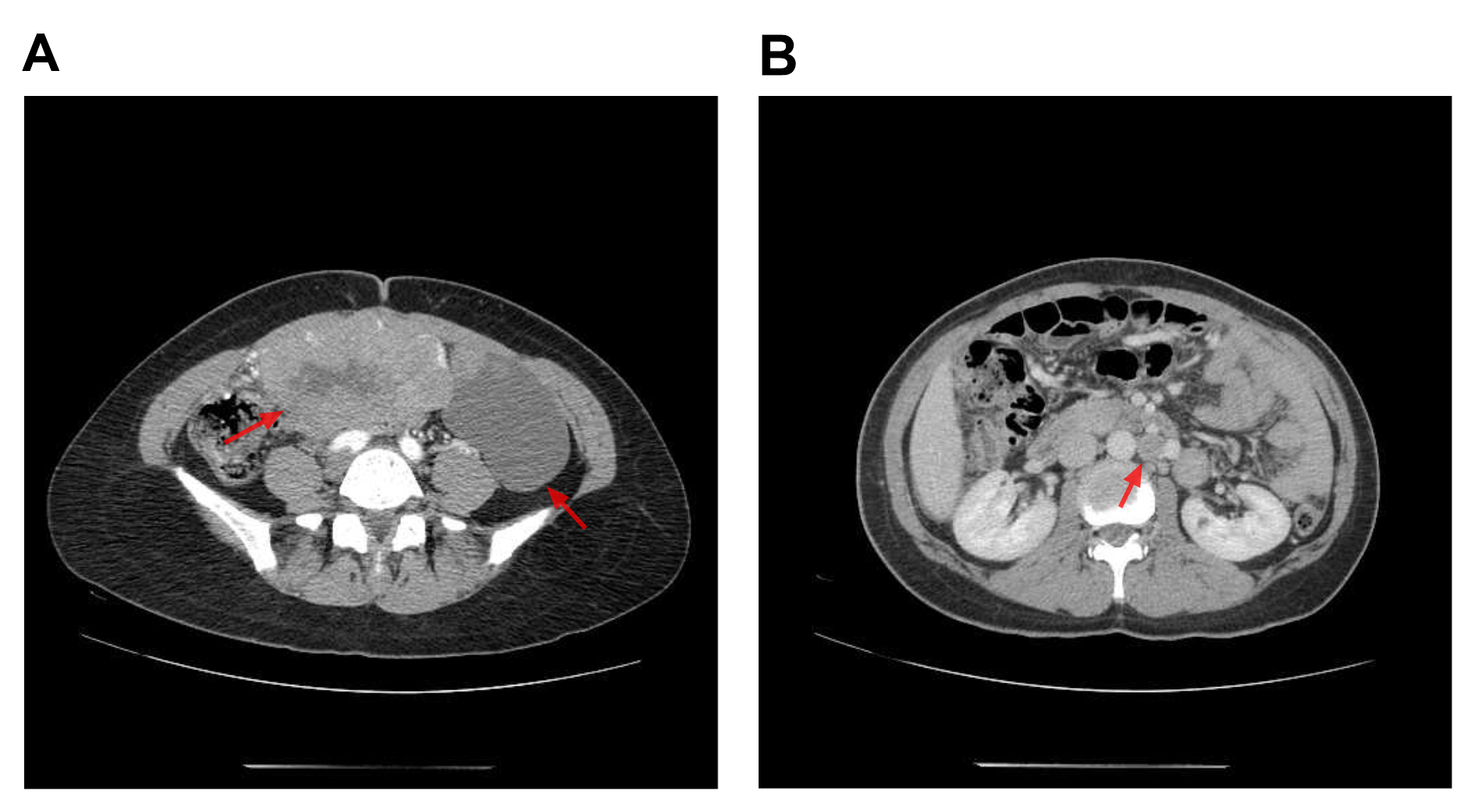

Supplement: Supplementary Figure 1 — Contrast-enhanced computed tomography (CT) images of the patient's abdomen (Date: 2009-6-27). (A) Preoperative tumors in the abdominal cavity. Enhanced CT findings suggest a large cystic solid mass in the lower abdomen as well as an encapsulated effusion adjacent to the mass. (B) Preoperative enlarged para-aortic abdominal lymph nodes. Enhanced CT showed enlarged lymph nodes adjacent to the abdominal aorta. Intraoperative exploration here revealed two lymph nodes measuring approximately 2*1.5 cm. [file Image1.tif]

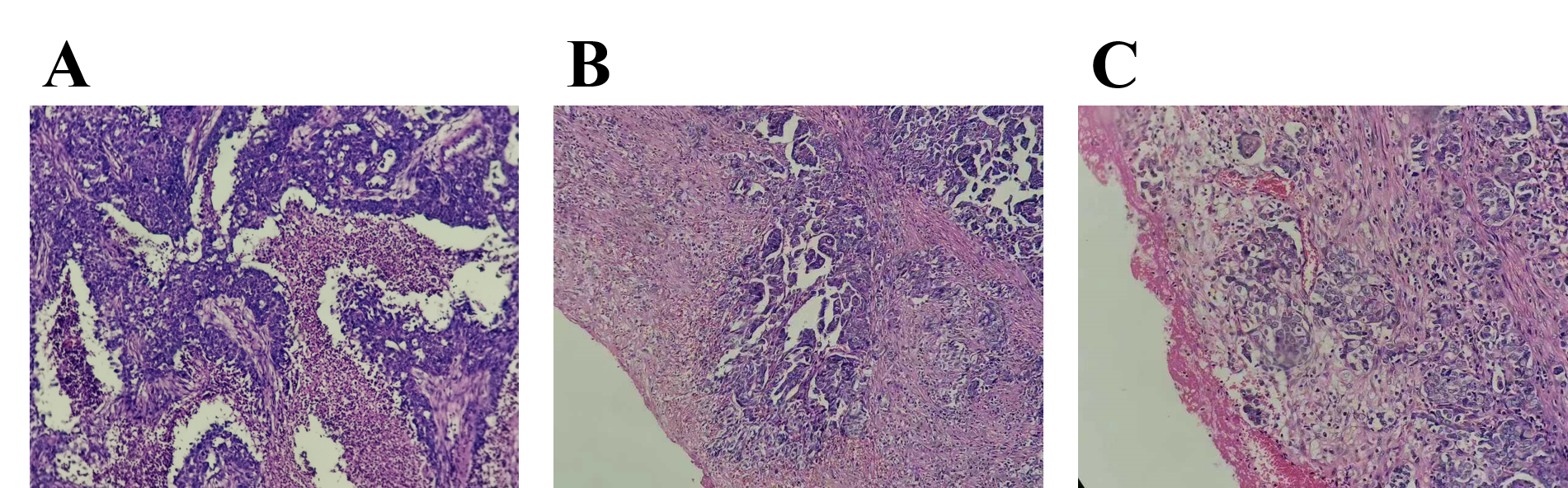

Supplement: Supplementary Figure 2 — Histopathologic findings of representative left ovarian high-grade serous ovarian cancer with multiple metastases (HE staining, ×200, scale bars: 200 µm). (A) Diffusely infiltrating tumor in the left ovary. (B) Metastatic tumor lesions in the mesentery. (C) Metastatic tumor lesions in the posterior peritoneum. [file Image2.tif]

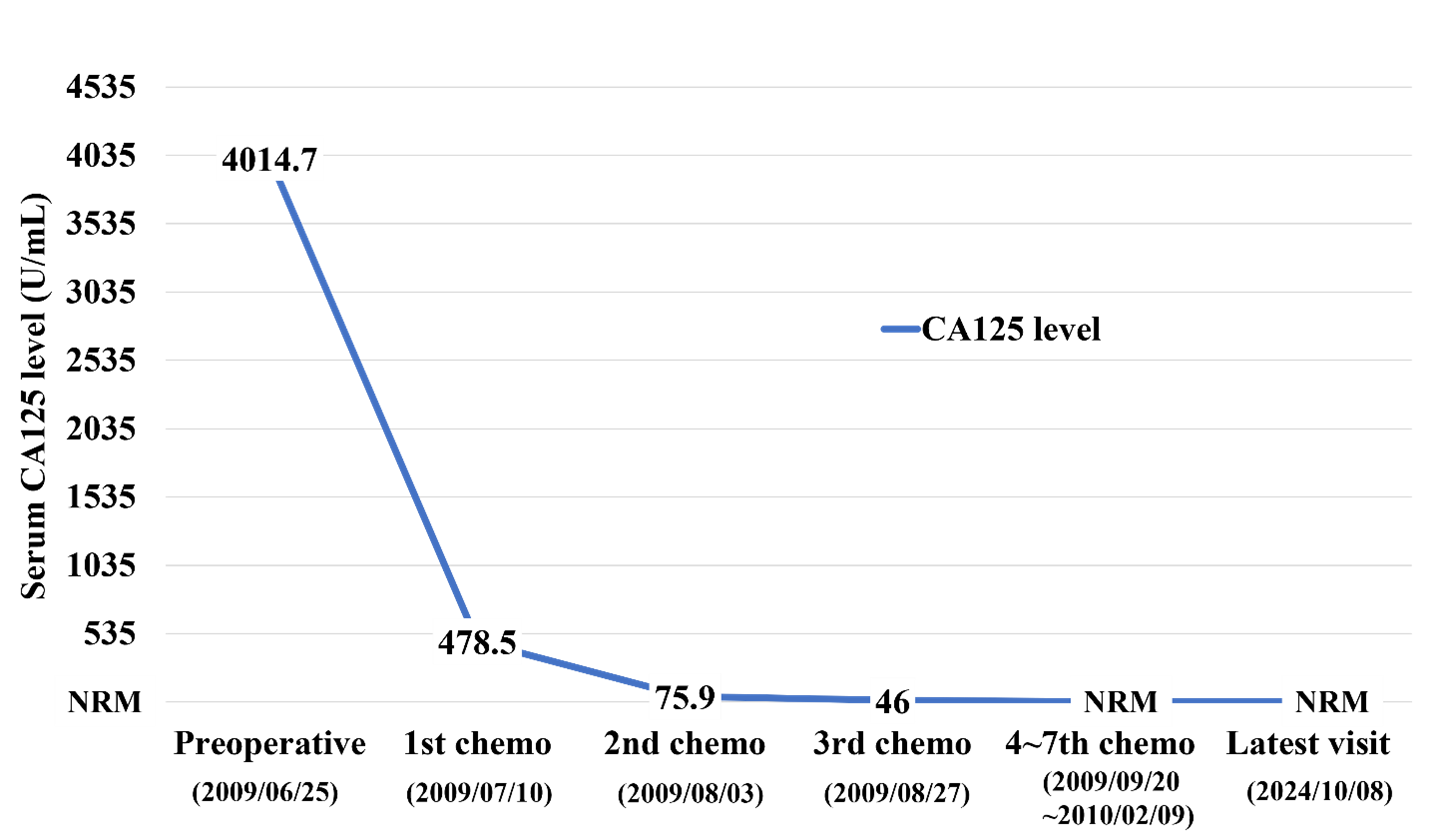

Supplement: Supplementary Figure 3 — Serum CA125 levels in this patient during the 15 years. The patient's CA125 level turned normal (reference value: <35 U/mL) after 3 chemotherapy treatments, and never elevated again during the subsequent 15 years of follow-up. (NRM=normal; 1st chemo= before the first chemotherapy). [file Image3.tif]

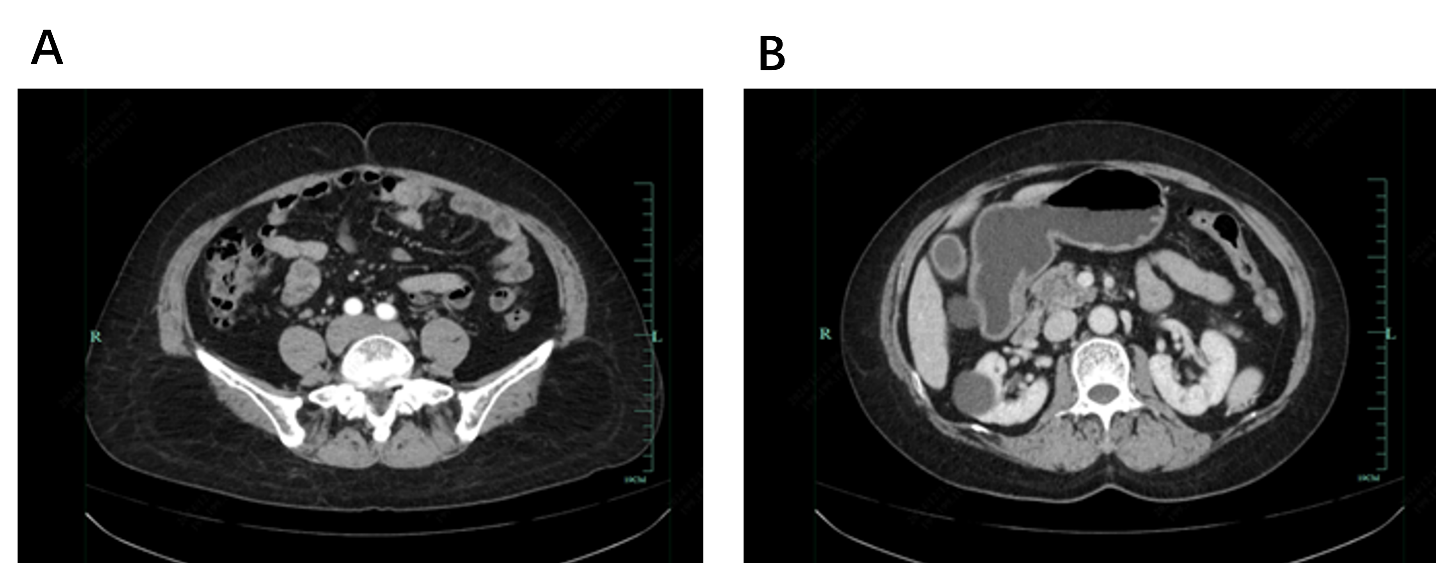

Supplement: Supplementary Figure 4 — Contrast-enhanced CT images of the patient's abdomen (Date:2024-10-10). (A) No tumour recurrence seen in the abdominal cavity. (B) Para-abdominal aortic lymph nodes are not enlarged. [file Image4.tif]
